# LPS-induced gene expression of inflammation-related genes in neutrophils from familial Mediterranean fever patients

**DOI:** 10.1186/1479-5876-10-S3-P56

**Published:** 2012-11-28

**Authors:** Gayane Manukyan, Eva Kriegova, Anush Martirosyan, Regina Fillerova, Maretta Tatyan, Martin Petrek

**Affiliations:** 1 Group of Molecular and Cellular Immunology, Institute of Molecular Biology, National Academy of Sciences, Yerevan, Armenia; 2 Laboratory of Immunogenomics and Proteomics, Medical Faculty, Palacky University, Olomouc, Czech Republic

## Background

Autoinflammatory disorders, such as Familial Mediterranean fever (FMF), characterized by abnormally increased inflammation, and mediated predominantly by the cells of innate immune system. FMF characterized by the episodes of self-resolving severe inflammation, with fever and serositis. The major cell type found in FMF inflammatory exudates are neutrophils. It is not known whether bacterial lipopolysaccharide (LPS) could influence neutrophil activation in FMF. We examined *in vitro* effect of LPS (10 ng/ml) exposure on expression of 13 selected genes in FMF and control neutrophils using quantitative real-time RT-PCR.

## Results

LPS exposure induced basal expression of TLR4 (4.4-fold increase, p<0.05), IL-1β (9-fold, p<0.01), IL-8 (13-fold, p=0.06), TNFAIP6 (11-fold, p<0.05) genes in FMF neutrophils, and of TLR4 (17.6-fold, p<0.001), IL-1β (25-fold, p<0.001), IL-8 (36-fold, p<0.001), TNFAIP6 (61-fold, p<0.001) in healthy neutrophils.

## Conclusions

Our findings indicate that gene expression in LPS-exposed FMF and control neutrophils is characterized by a number of shared alterations. In spite of pre-activated state of FMF neutrophils, LPS exposure further enhanced expression of the investigated genes. The ability of FMF neutrophils to enhance LPS-induced inflammatory reaction was however attenuated compared to healthy neutrophils. In conclusion, our data indicate that FMF neutrophils may display features of LPS tolerance, and this speculation should be explored further.

## Acknowledgements

Grant support: IGA_PU_LF_2012_007 and ANSEF NS_1987

